# Cytoplasmic expression of HuR may be a valuable diagnostic tool for determining the potential for malignant transformation of oral verrucous borderline lesions

**DOI:** 10.3892/or.2014.3017

**Published:** 2014-02-10

**Authors:** UMMA HABIBA, TETSUYA KITAMURA, AYA YANAGAWA-MATSUDA, KYOKO HIDA, FUMIHIRO HIGASHINO, YOICHI OHIRO, YASUNORI TOTSUKA, MASANOBU SHINDOH

**Affiliations:** 1 Department of Oral Pathology and Biology, Hokkaido University Graduate School of Dental Medicine, Kita-ku, Sapporo 060-8586, Japan; 2 Department of Vascular Biology, Hokkaido University Graduate School of Dental Medicine, Kita-ku, Sapporo 060-8586, Japan; 3 Department of Oral and Maxillofacial Surgery, Hokkaido University Graduate School of Dental Medicine, Kita-ku, Sapporo 060-8586, Japan

**Keywords:** oral verrucous lesion, HuR protein, molecular diagnosis

## Abstract

Oral verrucous carcinoma (OVC) is a low grade variant of oral squamous cell carcinoma, and oral verrucous hyperplasia (OVH) is a benign lesion without malignant features. However, pathologists are sometimes presented with borderline lesions and are indecisive as to diagnose them as benign or malignant. Thus, these lesions are tentatively termed oral verrucous lesions (OVLs). HuR is an ARE mRNA-binding protein, normally localized in the nucleus but cytoplasmic exportation is frequently observed in cancer cells. The present study aimed to elucidate whether expression of the HuR protein facilitates the diagnosis of true malignant lesions. Clinicopathological features were evaluated, and immunohistochemical analysis for p53, Ki67 and HuR proteins was performed in 48 cases of OVH, OVC and OVL, and the outcomes were correlated using appropriate statistical analysis. The association of these three proteins in relation to malignant transformation was analyzed after a 3-year follow-up of 25 OVL cases. The basal characteristics (age, gender and location) of all cases had no significant association with the types of lesions. Gingiva (39.4%) was the common site for all lesions. Distribution of the examined proteins had a significant association with the lesions. As compared with the OVLs, the number of immunostained-positive cells was significantly higher in the OVCs and lower in the OVH cases. During follow-up, 24% of the OVLs underwent malignant transformation for which high HuR expression and a diffuse staining pattern in the epithelium were observed. Taken together, the high degree of HuR expression with diffuse staining pattern in the epithelium may be an effective diagnostic tool that determines the potential of OVLs for malignant transformation.

## Introduction

Verrucous carcinoma was first described by Ackerman as a variant of squamous cell carcinoma of the oral cavity (1). Clinically, it is usually a warty, papillary exophytic, firm, non-ulcerating lesion with a broad base and a red, white, or variegated red and white surface (2). Histologically, it is characterized by verrucous proliferation of the squamous epithelium with wide and elongated rete ridges exhibiting a pushing border invasion into the underlying connective tissue with epithelial dysplasia. A mixed chronic inflammatory cell infiltrate composed of lymphocytes, plasma cells and histiocytes may also be prominent in the stroma (3,4). Oral verrucous hyperplasia (OVH) is a benign lesion without malignant features and cellular atypia. A properly oriented histological section including normal margin tissue is used for the morphological differentiation of OVH and oral verrucous carcinoma (OVC). In addition, immunohistochemistry such as p53, which is used to determine expression of mutant p53 protein, and Ki67, a proliferating marker, are used to diagnose OVC. Positive signals for p53 and Ki67 are usually increased in carcinoma specimens including OVC, whereas OVH alone does not show significant positive signals for these proteins. Pathologists are sometimes presented with specimens in which a clear distinction between the benign or malignant state is difficult to determine, and these are tentatively termed as oral verrucous borderline lesions (OVLs). The presence of some degree of epithelial dysplasia along with fewer positive signals of p53 and Ki67 protein makes it difficult to distinguish these lesions. Moreover, they share certain morphological similarities both clinically and histopathologically. However, it is vitally important to distinguish between OVC and its morphologically similar benign lesions considering the significance of therapeutic and prognostic implications of such lesions.

HuR is a ubiquitously expressed mRNA-binding protein. Intracellularly, HuR is localized predominantly in the nucleus but it shuttles between the nucleus and the cytoplasm. The export of HuR is mediated by its association with transportin 1 (Trn1) and transportin 2 (Trn 2) via the shuttling sequence termed’HNS’ in the hinge region and by its association with pp32, APRIL and SET α/β protein, which includes the nuclear export signal recognized by the export receptor chromosome maintenance region 1 (CRM1) (5–7). AU-rich elements (ARE) are located in the untranslated regions of many proto-oncogenes, growth factors and cytokine mRNAs as the core sequence of AUUUA. HuR binds to AREs to protect ARE-mRNAs against rapid degradation. Owing to nucleocytoplasmic translocation of HuR being necessary for its activity and the cytoplasmic presence of HuR found in several carcinomas, it has been hypothesized that cytoplasmic HuR expression may be a prognostic marker in cancer patients (8,9). Our previous study showed that export of HuR may be used as a diagnostic marker for oral cancers (10). In the present study, we analyzed the expression of p53, Ki67 along with HuR proteins in 48 cases diagnosed as OVH, OVC or OVL and compared them with clinicopathological parameters. The OVL cases were further investigated to determine the association of protein expression and malignant transformation. Thus, we aimed to evaluate whether the cytoplasmic expression of HuR protein facilitates the differentiation of true malignant lesions among OVLs.

## Materials and methods

### Patients and clinicopathological investigation

The present study examined 6 cases of OVH, 17 cases of OVC and 25 OVLs. All samples were collected from Hokkaido University Hospital between 1985 and 2010. Diagnosis was based on histological examination. Cases with epithelial verrucous hyperplasia without cellular dysplasia were diagnosed as OVH whereas OVCs were characterized by verrucous proliferation of squamous epithelium with wide and elongated rete ridges exhibiting a pushing border invasion into the underlying connective tissue with epithelial dysplasia. However, for OVLs we used the following diagnostic criteria: epithelial hyperplasia with hyperkeratosis and a verrucous surface, non-invasion of the hyperplastic epithelium into the lamina propria compared with adjacent normal mucosal epithelium, and lesions with varying degrees of epithelial dysplasia. The cases with OVLs were further followed up for 3 years to evaluate their potential for malignant transformation. Detailed demographic and clinical data for the cases of OVH, OVC and OVLs are listed in Table I.

### Immunohistochemical analysis

**Figure 1:**
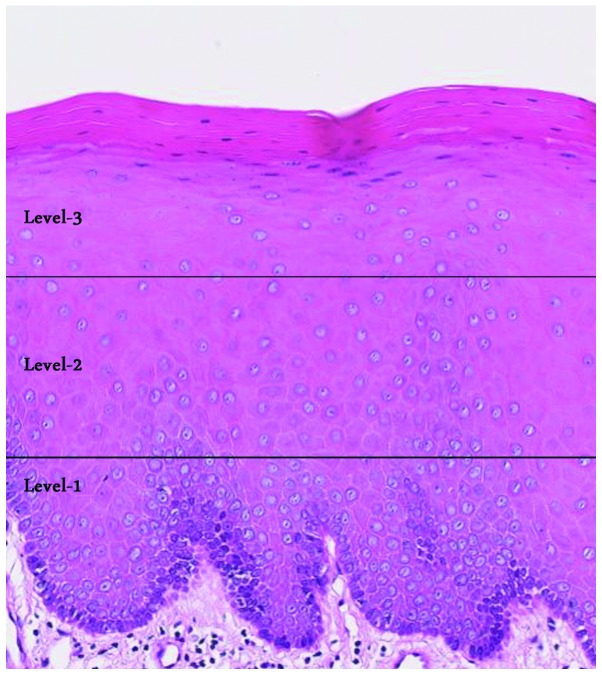
Imaginary division of an oral epithelium. The oral epithelium was divided into three levels to evaluate the distribution of the immunostained-positive cells. The lower one-third, lower two-thirds and upper one-third of the epithelium were designated as levels 1, 2 and 3, respectively.

The paraffin blocks of the specimens were cut in 5-μm sections and examined immunohistochemically. Sections were deparaffinized in xylene, rehydrated in graded alcohol and subjected to antigen retrieval by heat treatment in Tris-EDTA (TE) buffer. To inhibit endogenous peroxidase activity, the slides were then immersed in 3% H_2_O_2_ for 5 min followed by blocking solution [1% BSA in phosphated-buffered saline (PBS)] for 30 min. The immunohistochemical detection of HuR was carried out using anti-HuR monoclonal antibodies (1:6,000 dilution; Santa Cruz, Santa Cruz, CA, USA) in PBS in blocking solution in a humidified chamber at 4°C overnight. The sections were then subjected to Simple Stain Max PO (M) (Nichirei Bioscience, Tokyo, Japan) at 37°C for 30 min. Careful rinses were performed with several changes of PBS between the stages of the procedure. Visualization was carried out using the ChemMate EnVision kit/HRP (Dako, Tokyo, Japan), and the sections were counterstained with hematoxylin. The same tissues were immunostained with monoclonal antibodies against p53 and Ki-67 (1:100). The stained slides were examined by light microscopy, and the positive cell distribution in the different levels of the epithelium was observed. As shown in Fig. 1, the epithelium was divided into 3 levels: level 1 (lower one-third of the epithelium), level 2 (lower two-thirds of the epithelium) and level 3 (extending to the upper one-third of the epithelium).

Nuclear staining was considered as positive for p53 and Ki67 proteins, whereas cytoplasmic staining was considered as positive for HuR. The sections were initially scanned at low power, at least 3 high-power fields were then chosen randomly, and at least 1,000 cells were counted in level 1 of the epithelium for each case. The labeling indices (LIs) of cytoplasmic or nuclear staining of each antibody were defined as a ratio of immunostaining-positive cells to the total number of cells counted. Normal oral epithelium (NE) and oral squamous cell carcinoma (SCC) were used as negative and positive controls, respectively.

### Statistical analysis

The Chi-square test was applied to compare the association of the patient basal characteristics (age, gender, lesion location) and the distribution pattern of the target proteins (HuR, Ki67 and p53) in all lesions (OVH, OVC and OVL). The mean LIs of the three examined proteins were compared by the Student’s paired t-test. The association between the expression of proteins and malignant transformation of 25 OVLs was also assessed by Chi-square analysis. SPSS for Windows release 17.0 (SPSS) was used for statistical analysis. A value of P<0.05 was considered to indicate a statistically significant result.

## Results

**Figure 2:**
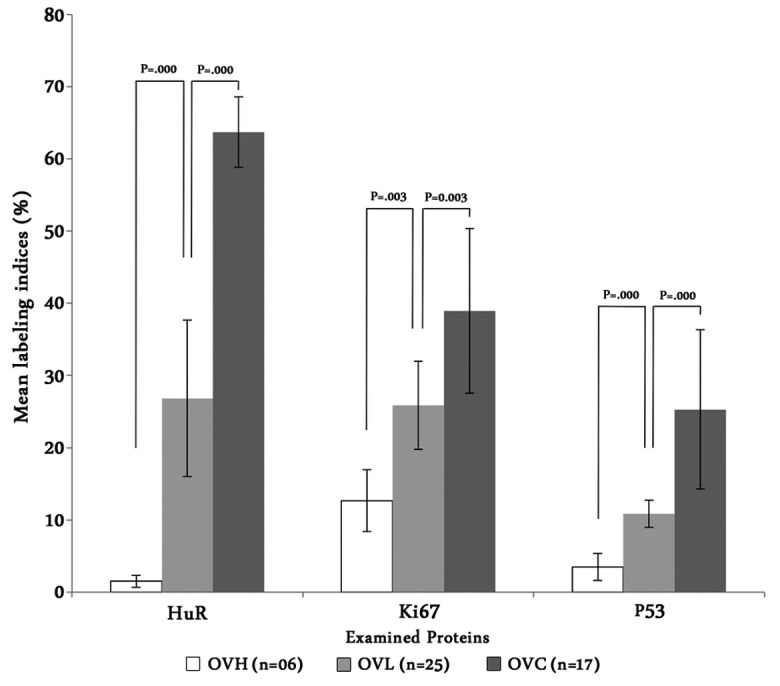
The mean labeling indices (LIs) (%) of the examined proteins. The ratio of immuostained-positive cells for p53, Ki67 or HuR to the total number of cells counted was evaluated for each case of oral verrucous hyperplasia (OVH), oral verrucous carcinoma (OVC) and oral verrucous lesion (OVL). The mean LI for the respective lesions was calculated and compared. Bars show standard deviation (SD).

**Figure 3:**
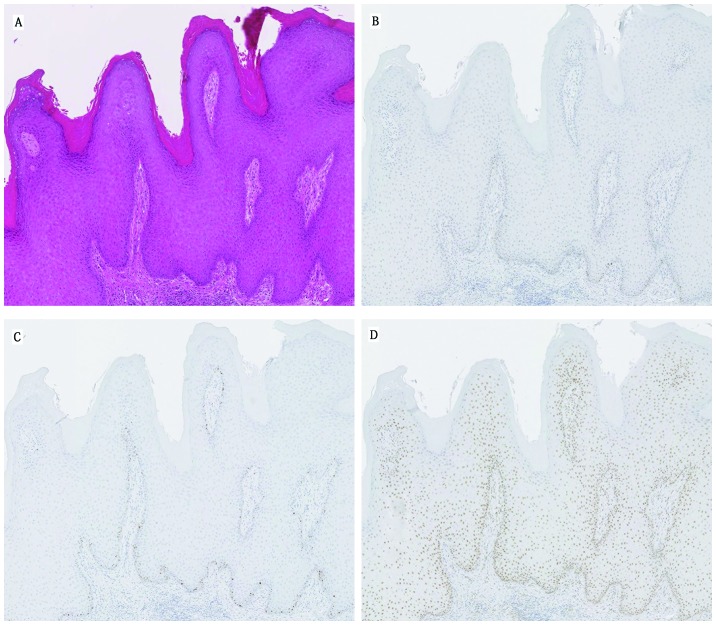
Expression of p53, Ki67 and HuR in oral verrucous hyperplasia (OVH). (A) Verrucous proliferation of squamous epithelium without dysplasia (H&E). A few positive cells are noted for (B) p53 and (C) Ki67 in basal to suprabasal layers of the epithelium. (D) HuR is detected in the nuclei of epithelial cells and cytoplasmic expression is rarely observed (A–D, magnification ×10). H&E, hematoxylin and eosin.

**Figure 4:**
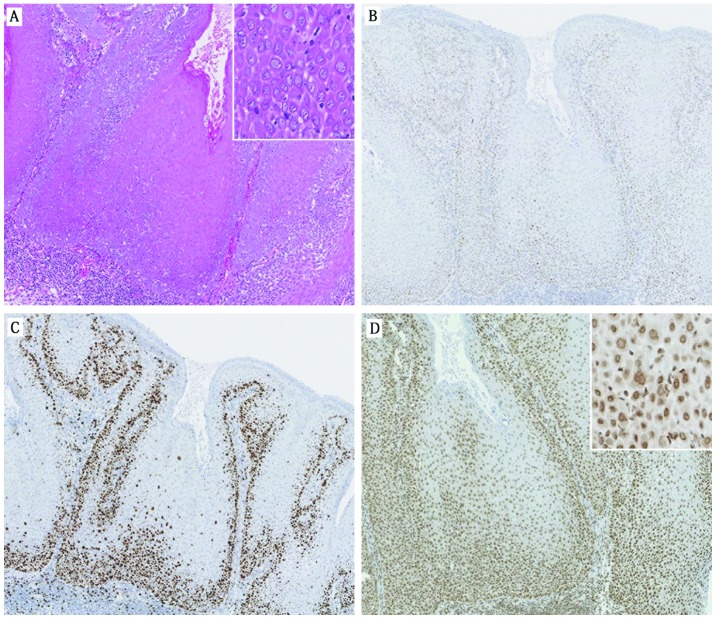
Expression of p53, Ki67 and HuR in oral verrucous carcinoma (OVC). (A) Atypical cells are prominent in the multi-layered basal cells (H&E). (B) p53- and (C) Ki67-positive cells are increased in number and noted in the middle of the epithelium. (D) Cytoplasmic HuR expression is commonly observed in lower two-thirds of the epithelium (A–D, magnification ×10). H&E, hematoxylin and eosin.

**Figure 5:**
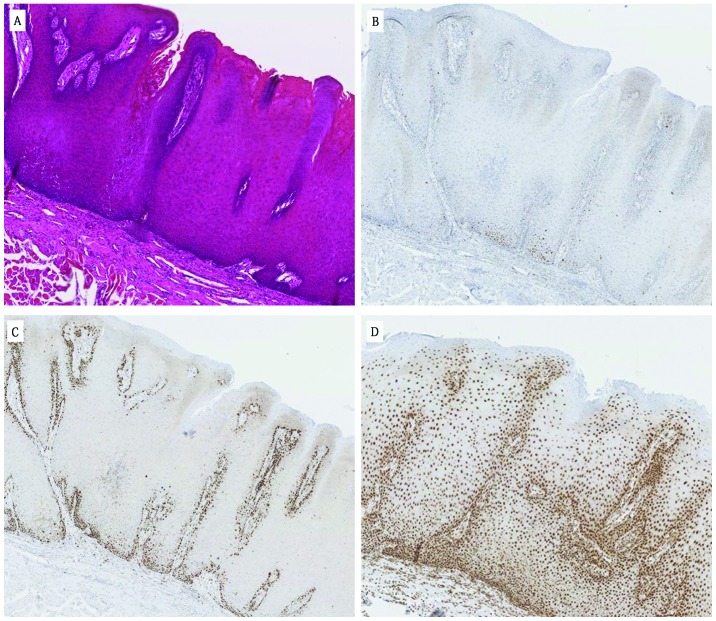
Expression of p53, Ki67 and HuR in oral verrucous lesion (OVL). (A) Basal cells are multi-layered and atypical cells are noted (H&E). However, the number of (B) p53- and (C) Ki67-positive cells are slightly increased and restricted to the lower third of the epithelium. (D) Cytoplasmic HuR expression is more evident than that in the OVH (A–D, magnification ×10). H&E, hematoxylin and eosin; OVH, oral verrucous hyperplasia.

The basal characteristics (age, gender and lesion location) of 48 patients and their association with 3 different types of oral lesions (OVH, OVL and OVC) are presented in Table I. The age range of the patients with OVH, OVC and OVLs was 32–73 years (mean age, 51), 47–96 years (mean age, 64) and 23–95 years (mean age, 71), respectively. Gingiva was the common site for all lesions (39.6%) followed by the tongue (29.2%). The male to female ratios were 1:1.8 and 1:1.3 for OVC and OVLs, respectively. Both lesions had a female predilection; however, there was no statistically significant association between basal characteristics and types of oral lesions. Immunohistochemical findings are summarized in Tables II and III and illustrated in Figs. 2–5. Distribution of all the three examined proteins (HuR, Ki67 and p53) in the different levels of the epithelium had a significant association with the oral lesions (Table II). The major finding was that in all OVH cases, positive signals for all proteins were restricted to level 1 of the epithelium (Fig. 3 and Table II) whereas there was a general trend for a more diffuse staining pattern in OVCs compared to OVHs and OVLs. More specifically, expression of HuR (64.7%) and p53 (52.9%) proteins extended up to levels 3 and 2 of the epithelium, respectively (Fig. 4 and Table II). In addition, 64.7% of OVCs showed Ki67-positive signals in level 2 of the epithelium. All OVL cases predominantly showed positive staining with HuR (64%), Ki67 (80%) and p53 (100%) in level 1 of the epithelium (Fig. 5 and Table II). However, in some cases HuR (35.3%) and Ki67 (20%) expression was observed in level 2 of the epithelium.

**Figure 6:**
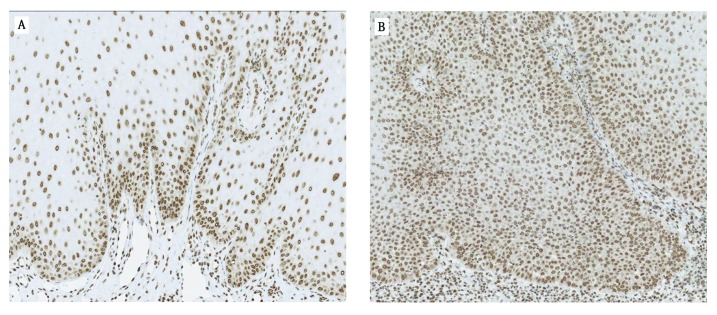
Comparison of HuR expression between the OVLs with low and high labeling indices (LIs). (A) In the low LI group (60% of cases), positive staining was noted in the lower one-third of the epithelium, whereas (B) in the high LI group (40% of cases) a positive signal extended to the lower two-thirds of the epithelium.

The mean LI (percentage of positive staining) of the examined proteins was analyzed in all lesions. The mean LI of the OVLs was significantly greater than that of the OVHs and lower than that of the OVCs (Fig. 2). The LI of HuR, Ki67 and p53 in the OVCs was 42.7-, 3.0- and 7.1-fold higher than the LI of the OVHs, and 2.4-, 1.5- and 2.3-fold higher than the OVL cases, respectively. When compared to the OVCs, only HuR expression markedly differed between the OVH and OVL cases. The OVL cases were also divided into two groups, high and low based on their mean LI for the respective protein (Table III). The mean LI for the HuR protein in the high group was 2-fold higher than that for the corresponding low group, whereas for Ki67 and p53 these differences were 1.6- and 1.3-fold, respectively. In addition, 90% of the cases having high HuR LIs showed a positive signal in level 2 of the epithelium (Table III). Expression of HuR in the high and low group cases is also displayed in Fig. 6. All 25 OVLs were further followed-up for 3 years to determine their risk to develop malignancy. Association between oral cancer development and expression of the examined proteins was analyzed and is presented in Table IV.

Six out of 25 (24%) OVLs underwent malignant transformation, and all of them had a high HuR LI (60%) and positive staining at level 2 (66.7%) of the epithelium (Table IV). This association was highly significant in terms of malignant transformation. However, no significant association was found when the LIs of Ki67 and p53 were analyzed. The lethal combination of the three examined proteins in relation to malignancy was also analyzed. As shown in Table IV, all of the cases that underwent malignant transformation had a high HuR LI in combination with either high or low Ki67 and p53. It is noteworthy that none of the cases having a low HuR LI transformed into malignancy. Consistent with these results, it is evident that OVLs with a high LI and wide distribution of HuR protein in the epithelium had a significantly higher oral cancer incidence than those with lower expression.

## Discussion

OVC and OVH are commonly diagnosed by morphological analysis, and expression levels of p53 and Ki67 are usually considered as biomarkers to facilitate the diagnosis of OVC. The p53 gene is reported to be the most frequent target for genetic alterations leading to cancer, and its mutation has been demonstrated in epithelial neoplasms. Although the p53 protein is expressed constitutively in all cells, it normally cannot be detected by immunohistochemical methods due to its short half-life and quick disintegration. Mutated p53 on the other hand, being more stable, accumulates in the cell and is readily detected by immunohistochemistry (11). Overexpression of p53 has been shown to occur in squamous premalignant lesions of the head and neck region, upper aerodigestive tract, the breast, and the urogenital tract, possibly in response to carcinogen-induced DNA damage (12–14). Our results are in agreement with previous studies, which demonstrated increased positivity for p53 protein (mean LI, 25%) extending up to the lower two-thirds of the epithelium (level 2), whereas OVH showed very few positive signals (mean LI, 3.5%) restricted to the basal cell layer of the epithelium (level 1).

Ki67 is a proliferation-associated protein that is believed to play a critical role in regulation of the cell cycle. The protein is expressed strictly during the active parts of the cell cycle, including the G1, S and G2/M phases, but not in resting cells in the G0 phase (15–17). Immunostaining with Ki67 highlights cells that are actively involved in proliferation. Positive expression of Ki67 has been used as a tool to estimate the proliferative potential in head and neck cancers and patient prognosis (18). Diffuse expression of Ki67 has been demonstrated throughout the entire thickness of the epithelium in invasive squamous carcinomas (19). Non-dysplastic acanthotic epithelium, in contrast, shows expression limited to the basal cell layer (20). In the present study, OVC cases showed increased positive staining (mean LI, 39%) extending to the lower two-thirds of the epithelium (level 2), whereas there were few positive cells in the OVH cases (mean LI, 13%) and they were localized in the lower one-third of the epithelium (level 1).

Oral verrucous borderline lesions (OVLs) are a much greater diagnostic and therapeutic challenge. Lesions showing mild to moderate dysplasia with few positive signals for p53 and Ki67 make diagnosis difficult, and pathologists often hesitate to categorize them as benign or already transformed malignant lesions considering the possible extensive surgical exposure of the cancer patient, whereas benign lesions are treated conservatively. In the present study, OVLs showed mild to moderate epithelial dysplasia (Fig. 5), and expression levels of Ki67 and p53 proteins (mean LI, 26 and 11%, respectively) were higher than OVHs but lower than OVCs. Analogous to OVHs, distribution of the positive cells was restricted predominantly to the lower one-third of the epithelium (level 1). In such borderline cases, morphological analysis and expression of p53 and Ki67 may not help to distinguish true malignant lesions or those that have the potential to transform into a malignancy.

It would be a great achievement in cancer diagnosis if we could evaluate the histochemical parameters for the risk of carcinoma in OVLs. Cytoplasmic HuR expression has been implicated in the malignancy of colon, ovarian, breast, salivary gland, uterine, larynx and prostate cancers and has been postulated to contribute to the cancerous malignant phenotype (21–25). Under physiological conditions, cellular stress induces HuR to bind AU-rich element (ARE)-containing mRNAs, and the complex is transported from the nucleus to the cytoplasm in a CRM1-dependent manner. In contrast, our previous study showed that HuR and ARE-mRNA in oral cancer cells are exported to the cytoplasm without CRM1 suggesting the possible role of cytoplasmic HuR expression in cell malignancy (10). In the present study, we investigated whether the cytoplasmic HuR expression pattern in OVLs may assist to discern true malignant lesions.

As expected, OVCs showed diffuse cytoplasmic expression of HuR (mean LI, 64%) throughout the epithelium, whereas expression was rarely noted in the OVHs (mean LI, 1.5%) and distributed in the lower one-third of the epithelium (level 1). Although HuR expression was restricted to level 1 in more than half (64%) of the OVLs, the expression was extended to the lower two-thirds (level 2) of the epithelium in the remaining 36% cases which is comparable to the expression of HuR in OVCs (35.3%). Notably, HuR expression in OVLs (mean LI, 27%) was 2.4-fold lower than that in the OVCs, whereas in OVHs the expression was 42.7-fold lower. This considerable difference between OVHs and OVLs compared to OVCs provide additional information regarding the role of HuR in cancer development of OVL cases.

We further followed up the OVLs to understand the risk of malignant transformation. All 25 OVL cases were divided into high and low group based on their LIs for the three examined protein, and their association with cancer development was assessed. As shown in Table IV, the OVL cases (6 out of 25) that were transformed into a malignancy had a high HuR LI and had a more diffuse staining pattern in the epithelium. None of the cases having a low HuR LI transformed into a malignancy. These observations are in line with the hypothesis that during tumorigenesis, HuR expression translocates from the nucleus to the cytoplasm. This specific finding indicates the need for proper treatment and careful follow-up for OVLs with high HuR expression. Our results suggest the strong possibility that expression of HuR in mild and moderate dysplasia may help to identify lesions with a high potential for malignant transformation, even when the positive signals for p53 and Ki67 are not significant. These findings indicate that the degree of HuR expression in OVLs may be an effective diagnostic factor that determines the potential of a lesion for malignant transformation. In conclusion, it is important to emphasize that appropriate diagnosis of OVLs can prevent wide surgical resection of the lesions as recommended for certain cases of oral well-differentiated squamous cell carcinoma.

## Figures and Tables

**Table I:** Demographic and clinical data of the patients with oral verrucous hyperplasia (OVH), oral verrucous carcinoma (OVC) and oral verrucous lesions (OVLs).

Characteristics	Total cases	Lesion types			P-value

		OVH	OVL	OVC	
All cases, n (%)	48	6 (12.5)	25 (52.1)	17 (35.4)	
Age (years)					0.586
Mean	65±17	53±15	68±17	65±16	
Median	69	51	71	64	
Range	23–96	32–73	23–95	47–96	
Gender, n (%)					0.774
Female	28 (58.3)	3 (10.7)	14 (50.0)	11 (39.3)	
Male	20 (41.7)	3 (15.0)	11 (55.0)	6 (30.0)	
Location, n (%)					0.196
Gingiva	19 (39.6)	2 (10.5)	7 (36.8)	10 (52.6)	
Tongue	14 (29.2)	2 (14.3)	10 (71.4)	2 (14.3)	
Buccal mucosa	5 (10.4)	1 (20.0)	4 (80.0)	0 (0.0)	
Lip	6 (12.5)	0 (0.0)	3 (50.0)	3 (50.0)	
Palate	3 (6.3)	1 (33.3)	0 (0.0)	2 (66.7)	
FOM	1 (2.1)	0 (0.0)	1 (100.0)	0 (0.0)	

**Table II:** Distribution of p53, Ki67 and HuR proteins in the different levels of the epithelium in the OVH, OVL and OVC cases.

		Protein expression in the different levels of the epithelium			
			
Examined proteins	No. of cases	Level 1	Level 2	Level 3	P-value
		
		n (%)	n (%)	n (%)	
HuR					0.000
OVH	6	6 (100)	-	-	
OVL	25	16 (64)	9 (36.0)	-	
OVC	17	-	6 (35.3)	11 (64.7)	
Ki67					0.002
OVH	6	6 (100)	-	-	
OVL	25	20 (80.0)	5 (20.0)	-	
OVC	17	6 (35.3)	11 (64.7)	-	
p53					0.000
OVH	6	6 (100)	-	-	
OVL	25	25 (100)	-	-	
OVC	17	8 (47.1)	9 (52.9)	-	

OVH, oral verrucous hyperplasia; OVL, oral verrucous lesion; OVC, oral verrucous carcinoma.

**Table III:** Categorization of the 25 oral verrucous lesions (OVLs) based on the labeling indices (LIs) of the examined proteins.

Examined proteins	Category	No. (%) of cases in the different categories (n=25)	Mean LI (%) in the different categories	No. (%) of cases with positive staining in the different categories	

				Level 1	Level 2
HuR	High LI (LI >27)	10 (40)	38.3±7.1	1 (10)	9 (90)
	Low LI (LI ≤27)	15 (60)	19.2±3.7	15 (100)	0 (0)
Ki-67	High LI (LI >26)	7 (28)	34.6±3.3	2 (29)	5 (71)
	Low LI (LI ≤26)	18 (72)	21.3±4.6	18 (100)	0 (0)
p53 High	LI (LI >11)	10 (40)	12.7±0.5	10 (100)	0 (0)
	Low LI (LI ≤11)	15 (60)	9.6±1.3	15 (100)	0 (0)

OVL cases were divided into two groups based on the mean labeling indices (LIs) for the respective protein. High and low groups represent the individual cases having LIs higher and lower than the mean LIs, respectively. OVLs, oral verrucous lesions.

**Table IV:** Association of the expression of the examined proteins and the development of malignancy in 25 oral verrucous lesions (OVLs).

Prognostic factors	No. of cases	Cases with malignant transformation	P-value

		n (%)	
Association with LIs of individual proteins			
HuR			0.001
High LI	10	6 (60.0)	
Low LI	15	0 (0.0)	
Ki67			0.169
High LI	7	3 (42.9)	
Low LI	18	3 (16.7)	
p53			0.702
High LI	10	2 (20.0)	
Low LI	15	4 (26.7)	
Association with combination of the examined proteins			
HH-KH-PH	5	2 (40.0)	0.005
HH-KL-PL	3	3 (100.0)	
HH-KH-PL	1	1 (100.0)	
HH-KL-PH	1	0 (0.0)	
HL-KL-PL	10	0 (0.0)	
HL-KH-PL	1	0 (0.0)	
HL-KH-PH	0	0 (0.0)	
HL-KL-PH	4	0 (0.0)	
Association with the distribution pattern of the examined proteins in the epithelium^a^			
HuR			0.000
Level 1	16	0 (0.0)	
Level 2	9	6 (66.7)	
Ki67			0.035
Level 1	20	3 (15.0)	
Level 2	5	3 (60.0)	

HH, HuR high; HL, HuR low; KH, Ki67 high; KL, Ki67 low; PH, p53 high; PL, p53 low; LIs, labeling indices.

a None of the proteins were expressed at level 3 of the epithelium. Since the p53 protein was expressed only at level 1 in all cases, statistical analysis was not possible.
